# Causal discovery approach with reinforcement learning for risk factors of type II diabetes mellitus

**DOI:** 10.1186/s12859-023-05405-x

**Published:** 2023-07-21

**Authors:** Xiu-E. Gao, Jian-Gang Hu, Bo Chen, Yun-Ming Wang, Sheng-Bin zhou

**Affiliations:** 1 College of Computer Science and Intelligent Education, Lingnan Normal University, Zhanjiang, 524048 Guangdong China; 2 College of Automation and Electrical Engineering, Dalian Jiaotong University, Dalian, 116028 Liaoning China; 3 College of Electronic and Electrical Engineering, Lingnan Normal University, Zhanjiang, 524048 Guangdong China

**Keywords:** Type 2 diabetes mellitus (T2DM), Risk factors, Reinforcement learning

## Abstract

**Background:**

Statistical correlation analysis is currently the most typically used approach for investigating the risk factors of type 2 diabetes mellitus (T2DM). However, this approach does not readily reveal the causal relationships between risk factors and rarely describes the causal relationships visually.

**Results:**

Considering the superiority of reinforcement learning in prediction, a causal discovery approach with reinforcement learning for T2DM risk factors is proposed herein. First, a reinforcement learning model is constructed for T2DM risk factors. Second, the process involved in the causal discovery method for T2DM risk factors is detailed. Finally, several experiments are designed based on diabetes datasets and used to verify the proposed approach.

**Conclusions:**

The experimental results show that the proposed approach improves the accuracy of causality mining between T2DM risk factors and provides new evidence to researchers engaged in T2DM prevention and treatment research.

## Introduction

Quality-of-life improvements and lifestyle changes have increased the proportion of diabetic patients worldwide annually. Diabetes has become an epidemic disease that seriously threatens human health [1–3], The risk factors of diabetes are of significant interest to medical professionals and researchers. These risk factors must be analyzed effectively. Currently, the related studies worldwide primarily focus on two issues: the discovery of new risk factors and the analysis of the relationships between risk factors.

### Discovery of new risk factors

In studies pertaining to different populations and ethnicities, many risk factors of diabetes have been identified. Studies [4, 5] confirmed the clinical value of glycated albumin through the diagnosis of diabetes mellitus. Tatsukawa et al. [6] discovered that the risk of diabetes in Asian populations presents a significant negative correlation with trunk fat and leg fat. Park et al. [7] identified body fat percentage (BF%) as a risk factor for type II diabetes mellitus (T2DM) in Koreans and discovered that an increasing BF% amplifies the risk of T2DM. Chen et al. [8]recognized “has_circ_CCNB1” and “has_circ_0009024” as potential risk factors of T2DM. Karamzad et al. [9] learned that the iron-regulating hormone/ferritin ratio is a highly predictive risk factor for T2DM. Ke et al. [10]demonstrated the positive correlation between maternal body mass gain during pregnancy and the risk of developing gestational diabetes mellitus. Shuping Zhang et al. [11] proved the positive correlation between the visceral adiposity index and T2DM occurrence among Chinese. The discovery of new risk factors can result in the early prevention of diabetes.

### Analysis of relationships between risk factors

The relationships between risk factors of diabetes have been primarily investigated via correlation analysis and causality analysis. Huang et al. [12] examined the interaction between biochemical markers in the development of diabetes. Zhu et al. [13] demonstrated that glycated hemoglobin is affected by visceral fat, total fat, total lean body mass, and the lean body mass of trunk and limbs. Bilal et al. [14] identified severe depression (SD) and perceived ethnic discrimination (PED) as risk factors for T2DM in sub-Saharan African migrants and reported that both SD and PED are positively associated with fasting blood glucose level. Wang et al. [15] constructed a causal prediction model for diabetes and studied the correlation among various risk factors, but did not reveal the causal relationship among risk factors. Wang et al. [16] revealed a potential causal relationship between abdominal obesity and hyperglycemia. Liu et al. [17] revealed the causal relationship between nonalcoholic fatty liver disease and central obesity. Previous analysis of the relationships among diabetes risk factors provides insight into the etiology and progression of diabetes.

The study of diabetes risk factors not only enhances the understanding of the pathophysiology of diabetes but also facilitates medical professionals in prescribing the appropriate medicine and reducing the side effects of medicines. Currently, statistical correlation analysis is the most typically used approach to investigate T2DM risk factors. However, this approach cannot readily reveal the causal relationships among risk factors and does not provide clinical decision-makers with the necessary causal knowledge. Moreover, the existing causal studies only elaborate the causal links between variables, i.e., intuitive descriptions and comprehensive analysis of their causal relationships are not provided.

**Fig. 1:**
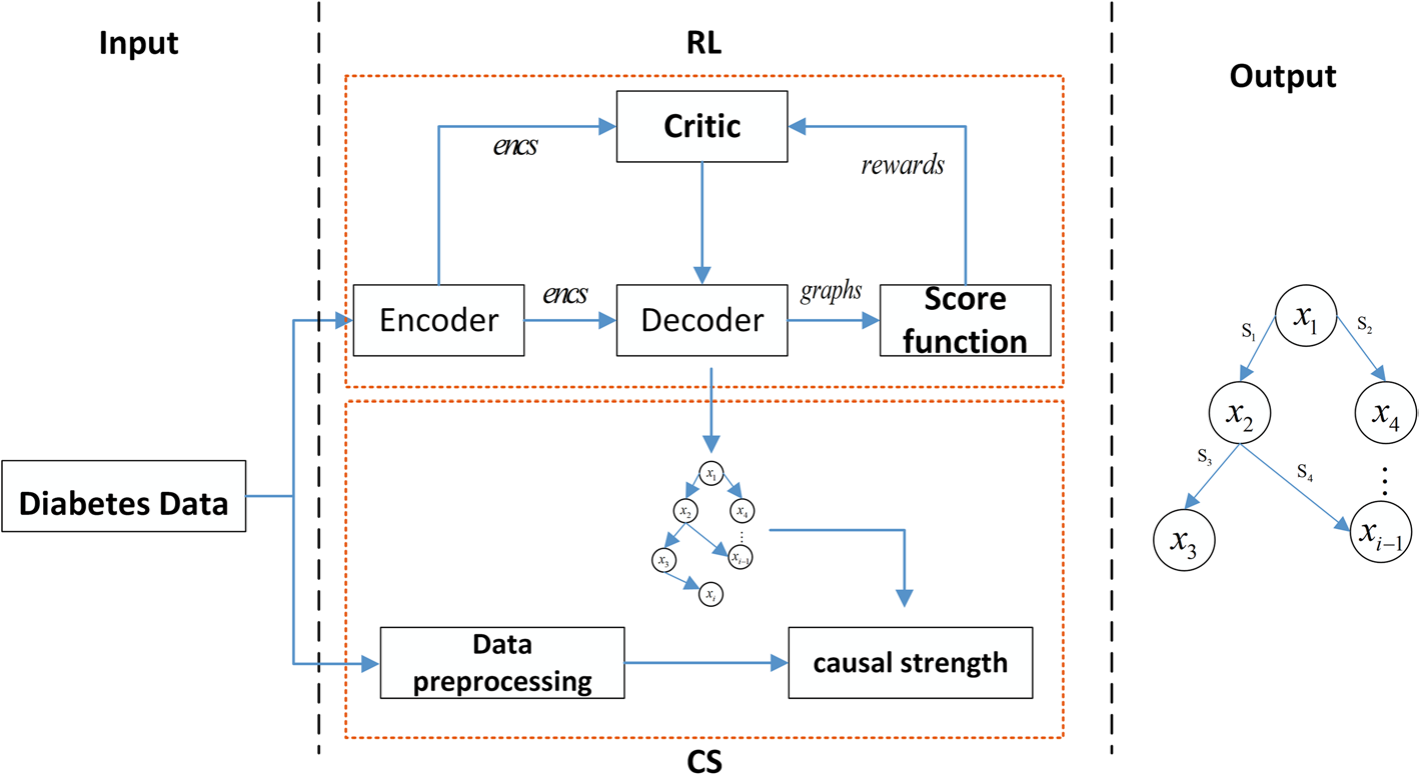
Structure of the proposed causal discovery approach with reinforcement learning for T2DM risk factors

Hence, a causal discovery approach with reinforcement learning for T2DM risk factors is proposed herein; the structure of the approach is illustrated in Fig. 1. The proposed approach yields the final causal structure of risk factors in two stages, namely, causal discovery and causal strength calculation [18, 19]. The skeleton of the causal structure is established using the causal discovery algorithm with reinforcement learning. Specifically, a directed graph is generated using an encoder–decoder model. Subsequently, the score function and two constraints of the graph are combined into a reward term to reinforce the acyclicity of the graph and to output the best return graph. Based on the skeleton of the causal structure, the causal strength is calculated as follows: the strength of each causal relationship is computed, and the complete causal structure is regarded as the final output. The proposed approach allows a more detailed description of the causal relationships between risk factors to be obtained and facilitates diabetes prevention and control research.

The contributions of this study are as follows:

1. A reinforcement learning model for T2DM risk factors is constructed.
2. A causal discovery algorithm for T2DM risk factors is designed.
3. Several experiments are designed using different diabetes datasets to verify the proposed causal discovery approach for T2DM risk factors.
4. The causal discovery results on different datasets are analyzed, and the causal relationships obtained using the proposed algorithm are shown to be reasonable.

The potential of our approach in mining the causal relationships between T2DM risk factors is confirmed based on a causal discovery model with reinforcement learning for risk factors, the process design of the causal discovery algorithm, and an experimental verification of the algorithm.

The remainder of this paper is organized as follows: “Methods” Section presents the causal discovery model with reinforcement learning for T2DM risk factors and the process involved in the causal discovery algorithm with reinforcement learning for T2DM risk factors. “Experiments” Section provides an analysis of the causal discovery algorithm for T2DM risk factors. “Discussion” Section discusses the experimental results based on the proposed causal approach. “Conclusions” Section provides a summary of the current research and recommendations for future research directions.

## Methods

### Algorithm principle

**Fig. 2:**
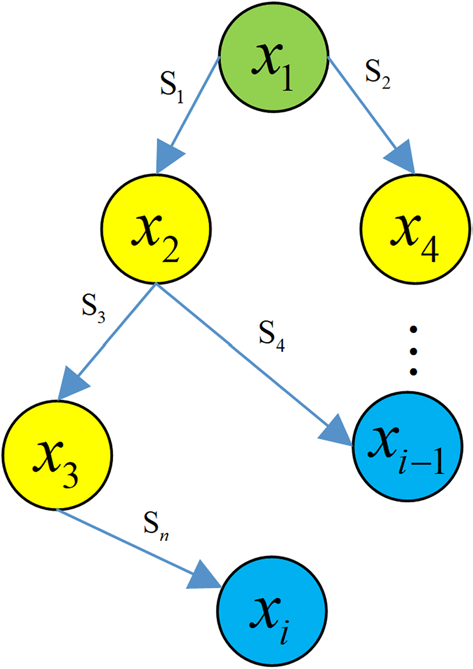
Causal structure simulated using observed data

As shown in Fig. 1, the proposed model comprises two stages, namely, causal discovery with reinforcement learning, and calculation of inverse information entropy (IIE) causal strength. The model input is the observed dataset $X=\left\{ x_{1},x_{2},x_3{},\ldots ,x_{i} \right\}$, where $x_{i}$ represents the dimension of the input observation data, and the model output is the causal structure composed of a causal graph *G* and causal strengths. Figure 2 shows the causal structure simulated by the observed data $O=\left\{ G,S\right\}$, where *S* denotes the strength of a causal relationship in graph *G*.

The model employs an encoder–decoder model to generate a directed graph. The encoder, which is the same as that in the original model [20], is composed of six identical encoding layers comprising two sublayers each. The first sublayer is a multi-headed self-attention network, and the second sublayer is a fully connected feedforward network arranged based on position. The sublayers are connected via residual connections. Finally, the output of each sublayer is normalized as follows:

1 $$\begin{aligned} Layer\,Norm(x+Sublayer(x)) \end{aligned}$$

where *Sublayer*(*x*) is a function realized by the sublayer. To ensure connectivity, the outputs of all sublayers and embedding layers in the model are of the same dimension $d_{model}=512$.

Considering the connection between different variables, a single-layer decoder is used.

2 $$\begin{aligned} g(W_1,W_2,u)=u^Ttanh(W_1enc_i+W_2enc_j) \end{aligned}$$

where ${W_1},{W_2} \in {\mathbb {R}^{{d_h} \times {d_n}}}$ and $u\in {\mathbb {R}^{{d_h} \times 1}}$ are trainable parameters, $d_h$ is the number of hidden layers associated with the decoder and $d_n$ is the dimension of the encoder output *encs*. To generate the adjacency matrix, each item is input to the sigmoid function and then sampled based on a Bernoulli distribution with probability $\sigma (g)$, which represents the probability of edges between variables.

3 $$\begin{aligned} M\sim Ber(\sigma (g)) \end{aligned}$$

At the same time, in order to avoid self-circulation of variables, the (*i*, *i*)th item in the adjacency matrix will be directly marked. When the encoder information of all variables is cyclically input, a complete directed graph adjacency matrix can be obtained.

The score function uses the Bayesian information criterion (BIC). Since the BIC score is uniquely decomposable, the penalty term can be adjusted when applying this score. The BIC score function on graph $\mathcal {G}$ can be expressed as follows:

4 $$\begin{aligned} {S_{BIC}}(\mathcal {G}) = - 2\log p(X;\mathop L\limits ^ \wedge ;\mathcal {G}) + {d_L}\log m \end{aligned}$$

where $\mathop L\limits ^\wedge$ is the maximum likelihood estimation, $d_L$ is the dimension of parameter *L* and *m* is the amount of data *X*.

To ensure that a directed acyclic graph is created, a score function and two acyclic constraints are incorporated into the reward and penalty terms.

5 $$\begin{aligned} reward = - [S_{BIC}(\mathcal {G}) + {\lambda _1}I(\mathcal {G} \notin DAGs) + {\lambda _2}h(A)] \end{aligned}$$

where $I(\cdot )$ is the indicator function, $\lambda _{1},\lambda _{2}$ are hyperparameters in model training, $A \in \{0,1\}^{d\times d}$ and *h*(*A*) is a function proposed by Zhang et al [21]. The binary adjacency matrix of a directed graph $\mathcal {G}$ is acyclic if and only if the following holds true:

6 $$\begin{aligned} h(A) = trace({e^A}) - d = 0 \end{aligned}$$

where $e^A$ is the matrix exponent of *A*.

The expected return on training can be expressed as follows:

7 $$\begin{aligned} J(\varphi |s) = \mathbb {E}_{A\sim \pi (\cdot |s)}\{ - [S_{BIC}(\mathcal {G}) + {\lambda _1}I(\mathcal {G} \notin DAGs) + {\lambda _2}h(A)]\} \end{aligned}$$

where $\pi (\cdot |s)$ and $\varphi$ are the strategy and neural network parameters for graph generation, respectively. During training, the input is constructed by obtaining random samples from the observed dataset *X*. The output of the encoder is imported to the critic, which is a simple two-layer feedforward neural network with the tanh function. The critic solves the mean squared error between the predicted and actual rewards and penalties, and is trained using the Adam optimizer.

Based on [19], the IIE causal strength can be expressed as follows:

8 $$\begin{aligned} T = \frac{1}{{|S({p_{{X_2}}}) - S({p_{{X_1}}})|}} \end{aligned}$$

where $S({p_{{x_1}}})$ and $S({p_{{x_2}}})$ are the information entropies of variables $x_1$ and $x_2$, respectively. For a finite point set, the entropy of the probability distribution for the risk factors can be estimated using the entropy estimator [22, 23] as follows:

9 $$\begin{aligned} \mathop S\limits ^ \wedge (X) = \psi (n) - \psi (1) + \frac{1}{{n - 1}}\sum \limits _i^{n - 1} {\log |{x_{i + 1}} - {x_i}|} \end{aligned}$$

where $\psi (n)$ is the double gamma function and *n* is the dimension of the variable diabetes data *X*.

Based on Eq. (8), the IIE causal strength is calculated using raw data. However, the calculated causal strength may deviate from the actual value owing to the dimensionality difference between variables in the raw data. Thus, the IIE causal strength of the normalized data is expressed as follows:

10 $$\begin{aligned} {T_N} = \frac{1}{{|S({p_{{X_2},N}}) - S({p_{{X_1},N}})|}} \end{aligned}$$

### Algorithm flow

The proposed causal discovery approach with reinforcement learning for T2DM risk factors comprises three stages, namely, data processing, causal discovery, and causal strength calculation.

**Fig. 3:**
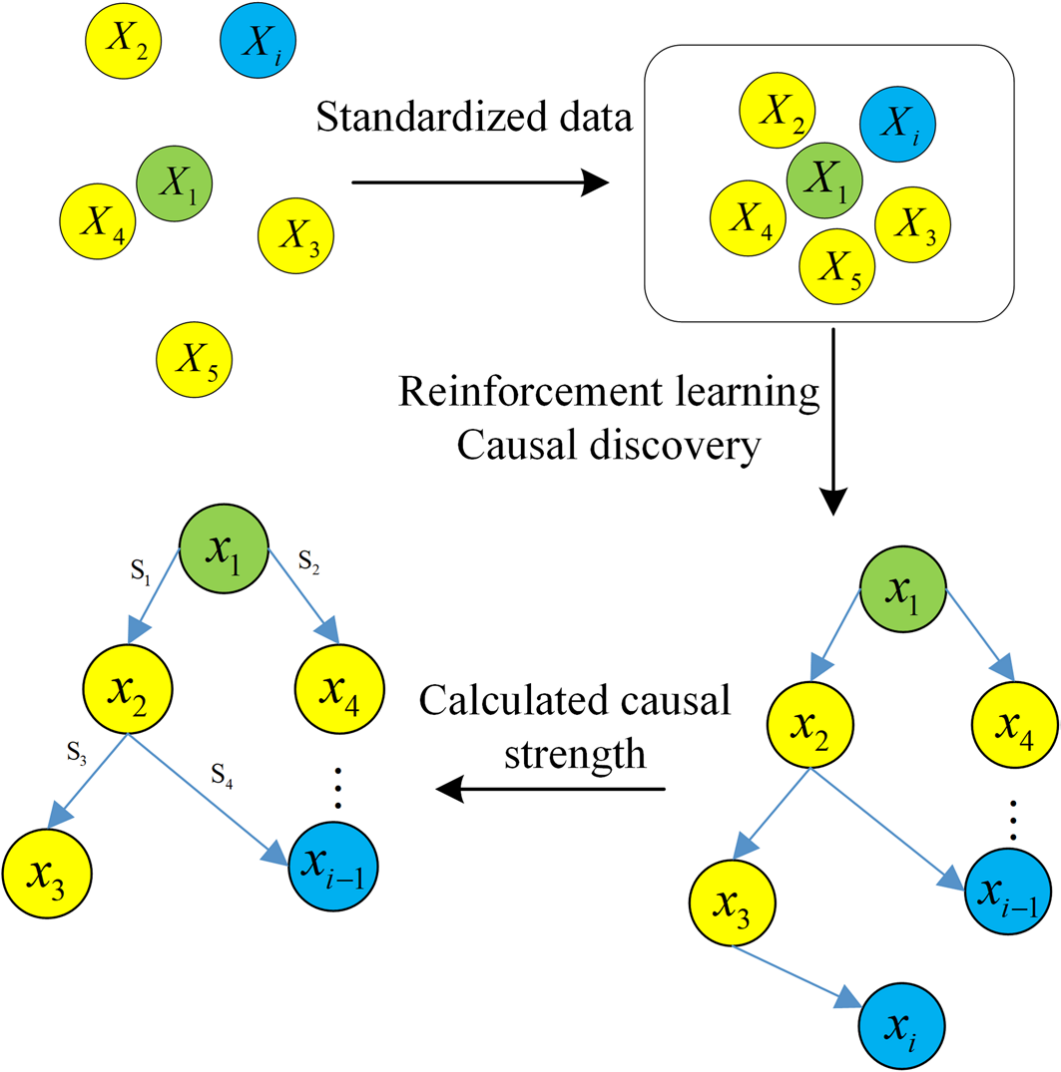
Process of proposed approach

As shown in Fig. 3, the proposed approach comprises the following steps:

*Step 1* Enter and normalize the observed data pertaining to diabetes risk factors.

*Step 2* Set hyperparameters such as number of iterations, model training device, and scoring function.

*Step 3* Import the normalized data to the encoder for encoding.

*Step 4* Import the outputs of the encoder and the critic to the decoder, which performs calculation using Eq. (2) and obtains samples based on a Bernoulli distribution with probability.

*Step 5* Rate the output directed graph using Eq. (3), return the rewards and penalties to the critic, and save the maximum reward and penalty.

*Step 6* Assess if the preset number of iterations is reached. If yes, output the directed graph of the maximum score; otherwise, return to Step 3.

*Step 7* Based on the output directed graph from Step 6, calculate the causal strength using the normalized data and remove redundant or wrong edges with causal strength of less than 0.5.

*Step 8* Output the final causal structure.

## Experiments

In order to verify the effectiveness of the reinforcement learning causal discovery method for T2DM risk factors proposed in this paper, two real data causal discovery simulations are designed in the experimental part. Through the experiment, the causal structure of the corresponding data can be clearly displayed, which is convenient for comparative analysis of the correctness of the generated causal structure.

### Experimental data

The experimental data were obtained from two sources: (1) Two Pima Indian diabetes datasets, which contained 768 [24] and 2000 [25] samples, separately (Note: All samples are female), The data comes from the Kaggle platform; (2) A diabetes dataset was synthetized from search results on the website of the National Health and Nutrition Examination Survey (NHANES) [26]. The acquisition process of the NHANES data set is briefly described as follows: First, the data from 2011 to 2020 were counted, and the data before 2011 were different from the current human body data, which was not representative, so the data before 2011 were excluded; at the same time, Due to the impact of the new coronavirus, there are many missing data after 2020, so the data after 2020 are also excluded; secondly, 16 kinds of physiological data that may be related to diabetes are downloaded, such as blood sugar concentration, glycosylated hemoglobin and BMI, etc.; again, according to the investigator number (SEQN), the data of the same person were integrated; finally, the data containing null and invalid values were deleted, and finally the NHANES data set with a sample size of 13921 was generated.

The Pima Indian diabetes datasets comprised eight variables: gravidity $X_1$, 2-h glucose level $X_2$, diastolic blood pressure (mm Hg) $X_3$, triceps skin fold thickness (mm) $X_4$, 2-h insulin level (mu U/mL) $X_5$, body mass index (BMI) $X_6$, diabetes pedigree function $X_7$, and age $X_8$. Among them, the diabetes pedigree function contained genetic information regarding the subject’s family history of diabetes.

The NHANES dataset included 13 variables: age $X_1$, race $X_2$, diastolic blood pressure $X_3$, body weight $X_4$, BMI $X_5$, albumin in urine $X_6$, creatinine in urine $X_7$, high-density lipoprotein $X_8$, triglycerides $X_9$, low-density lipoprotein $X_{10}$, glycated hemoglobin $X_{11}$, insulin $X_{12}$, and fasting glucose $X_{13}$.

### Experimental analysis

#### Pima Indian diabetes datasets

**Fig. 4:**
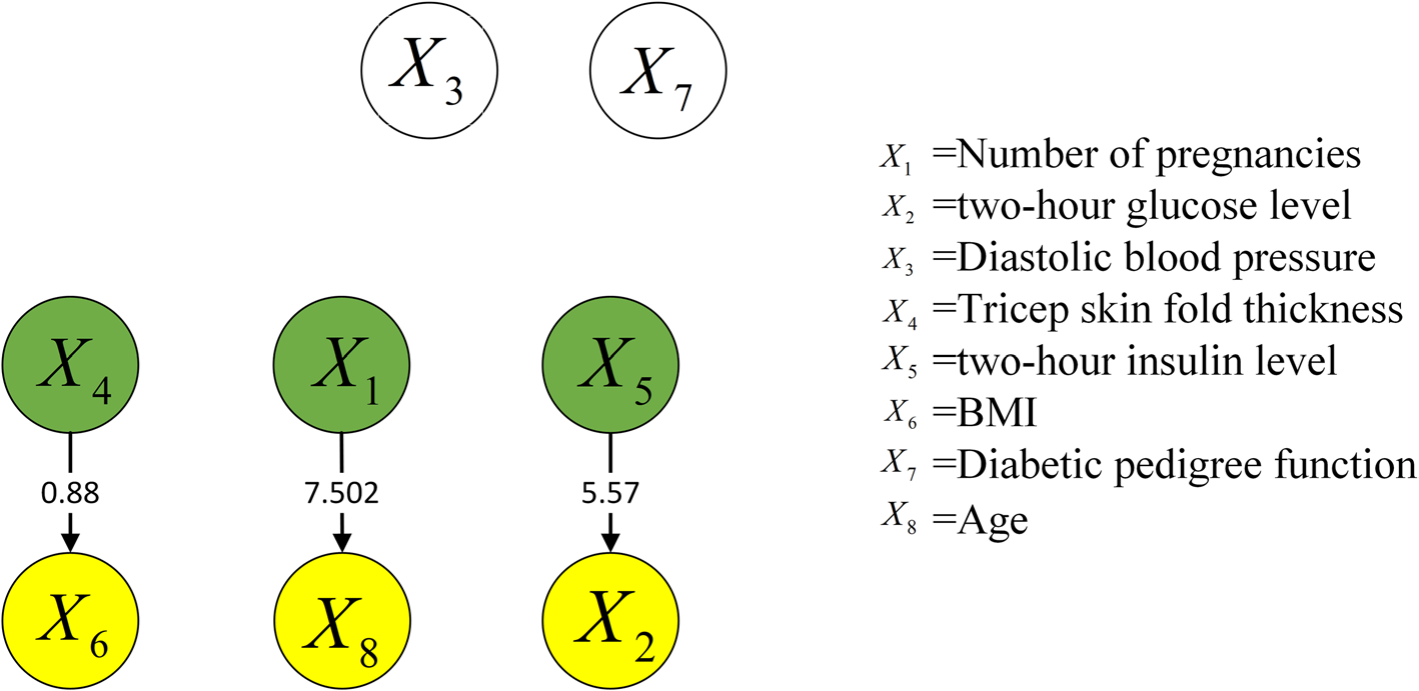
Causal structure of risk factors for 768 samples (normalized data)

**Fig. 5:**
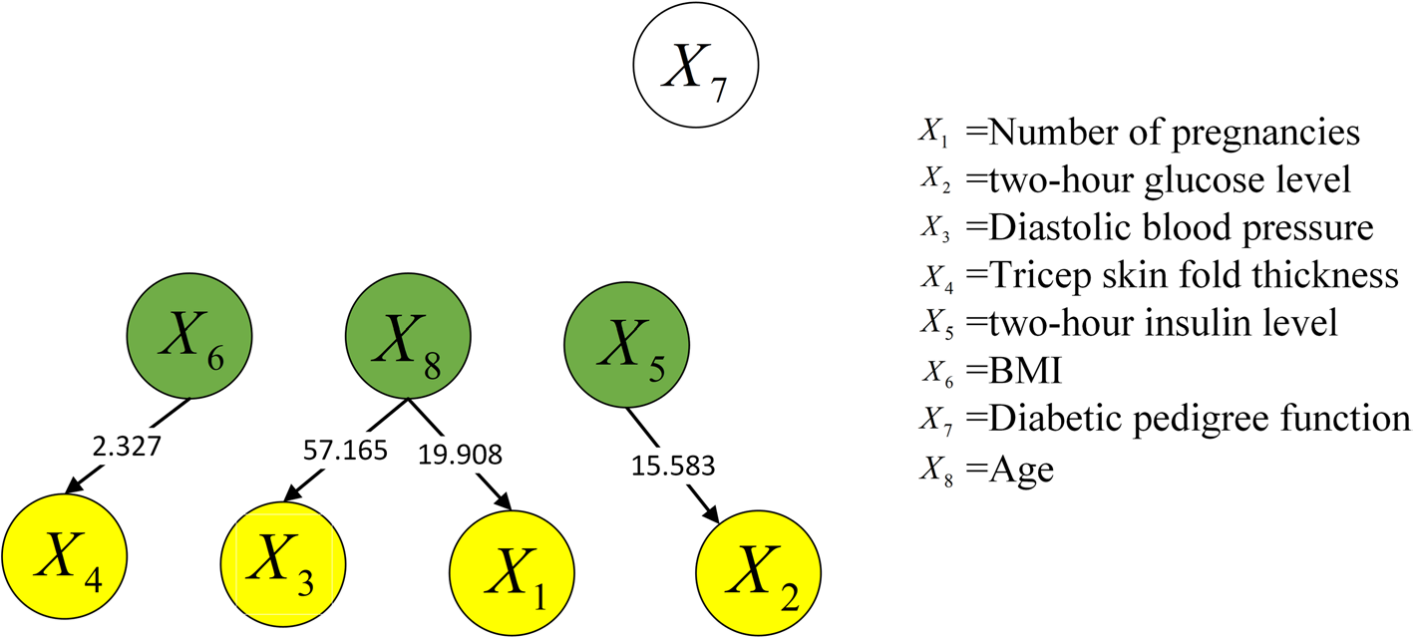
Causal structure of risk factors for 2000 samples (normalized data)

Figures 4 and 5 show the causal structures of risk factors in the two Pima Indian diabetes datasets, which contained 768 and 2000 samples, separately. The structures were obtained via the proposed approach, which normalizes the raw data in the first step. One identical causal relationship, i.e., ${X_5} \rightarrow {X_2}$, was shown in the two figures, which indicates that a change in the insulin level alters the plasma glucose level. Figure 5 shows an additional causal relationship, i.e., ${X_8} \rightarrow {X_3}$, which shows that a change in age alters the diastolic blood pressure. Furthermore, Figs. 4 and 5 show that two causal relationships between risk factors are changed: (1) the relationship between triceps skin fold thickness $X_4$ and BMI $X_6$; (2) the relationship between gravidity $X_1$ and age $X_8$. In addition, the diabetes pedigree function does not indicate a causal relationship with other variables.

Comparing the causal strengths in Figs. 4 and 5, the causal structure of the set containing 2000 samples generally exhibits a higher causal strength than that of the set containing 768 samples. This implies that a larger sample size stabilizes the causal structure of the risk factors more effectively. Moreover, the causal strength between a pair of variables tends to increase as the sample size increases from 768 to 2000. Thus, the causal relationships are more convincing in larger datasets.

**Fig. 6:**
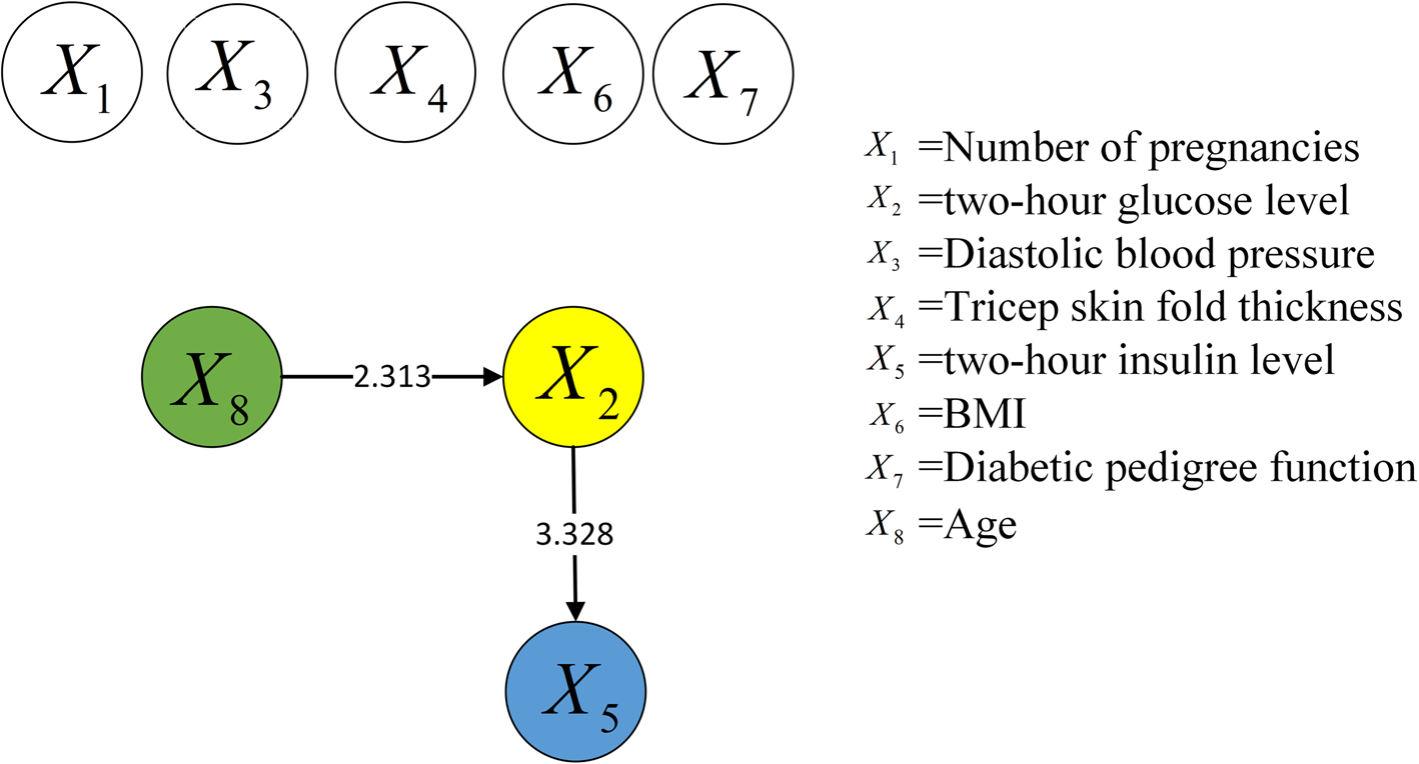
Causal structure of risk factors for 768 samples (raw data)

**Fig. 7:**
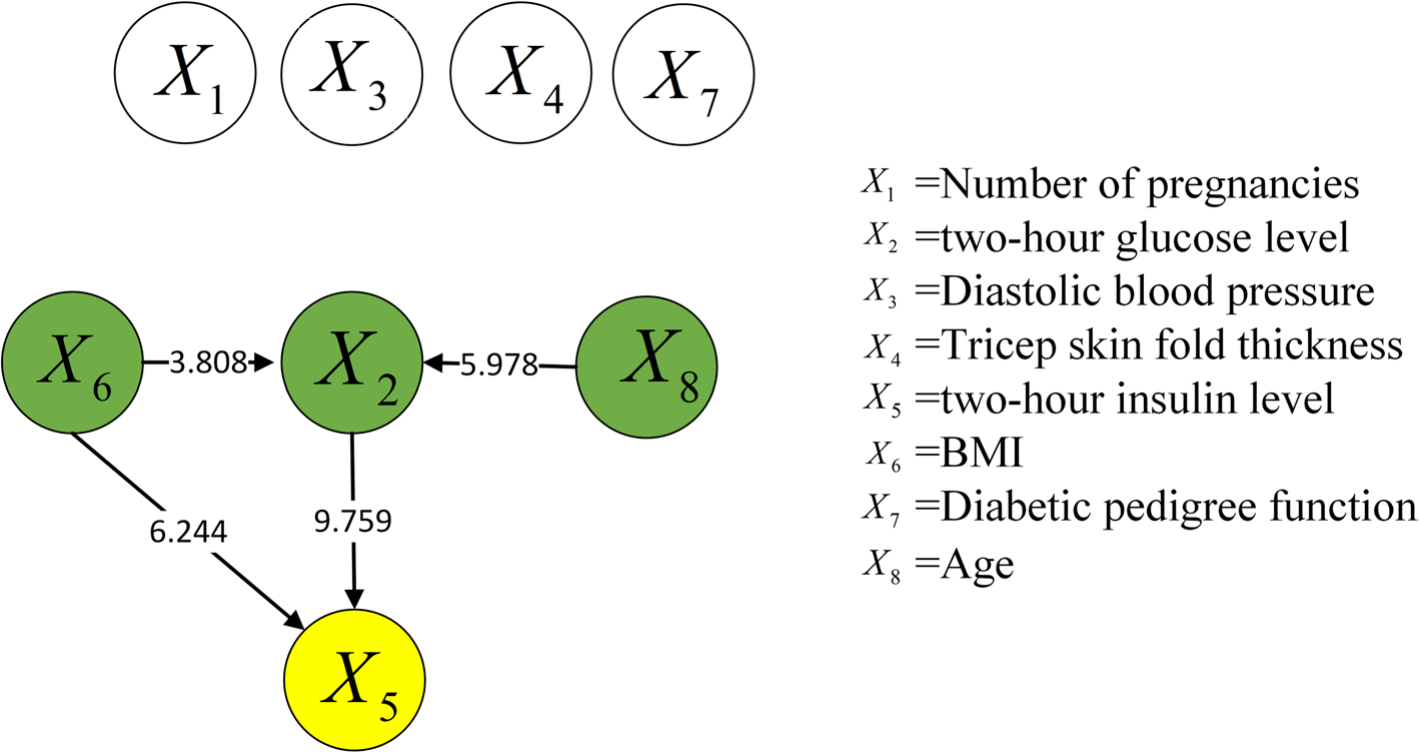
Causal structure of risk factors for 2000 samples (raw data)

To reveal the manner by which data normalization affects the causal discovery approach for risk factors, the same causal discovery experiment of risk factors was performed using the raw data. Figures 6 and 7 show the causal structures of risk factors in the two Pima Indian diabetes datasets, which contained 768 and 2000 samples, respectively. This time, the raw data were used directly without normalization.

Compared with Figs. 4 and 6 shows an additional causal relationship, i.e., ${X_8} \rightarrow {X_2}$, which suggests that age alters the blood glucose level. However, this is not confirmed theoretically. The two factors do not affect the diagnosis of diabetes. For children, adults, and senior citizens, the same criterion applies: the glucose level in blood is normal [27] when the fasting blood glucose level remains below 7.0 mmol/L. Therefore, the new causal relationship is incorrect. The other new causal relationship is ${X_2} \rightarrow {X_5}$, which indicates that an increase in the blood glucose level will alter the insulin level: the lower the blood glucose level, the higher is the insulin level. However, this trend is based entirely the regulation of the human body. Furthermore, the causal strength of ${X_5} \rightarrow {X_2}$ in Fig. 4 is 5.57, whereas that of ${X_2} \rightarrow {X_5}$ in Fig. 6 is 3.328. Thus, ${X_5} \rightarrow {X_2}$ is believed to be more accurate.

Similarly, ${X_8} \rightarrow {X_2}$ and ${X_2} \rightarrow {X_5}$ are indicated in Fig. 7 and indicate the same change laws as above, as compared with Fig. 5. Thus, ${X_8} \rightarrow {X_2}$ is deemed incorrect. Additionally, the causal strength of ${X_5} \rightarrow {X_2}$ is greater than that of ${X_2} \rightarrow {X_5}$. In addition, new relationships are indicated in Fig. 7, such as ${X_6} \rightarrow {X_2}$ and ${X_6} \rightarrow {X_5}$. Between them, ${X_6} \rightarrow {X_2}$ indicates that BMI affects the blood glucose level; however, this causal relationship has no scientific basis. As stated above, the same criterion for diabetes diagnosis based on blood glucose level applies to different groups of people. Hence, ${X_6} \rightarrow {X_2}$ is considered an incorrect causal relationship. Meanwhile, ${X_6} \rightarrow {X_5}$ suggests that BMI results in insulin changes. Some studies [28, 29] indicated a significant correlation between insulin resistance and obesity; however, the causality must be further investigated.

Based on the analysis above, the causal structures in the raw datasets contain numerous incorrect and unknown relationships, whereas those in the normalized datasets contain more causal relationships with higher accuracies. Therefore, the accuracy of causal discovery can be effectively improved by normalizing the raw dataset in advance.

#### NHANES dataset

**Fig. 8:**
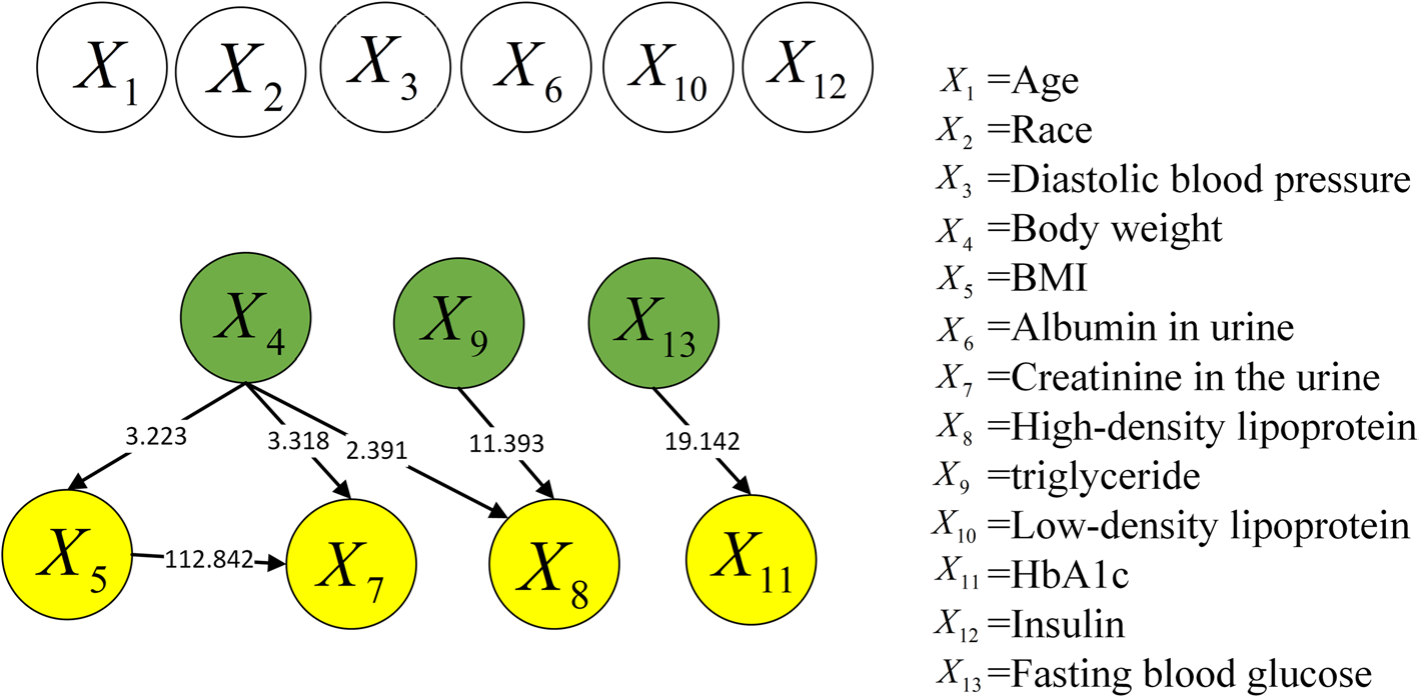
Causal structure of NHANES dataset (normalized data)

Figure 8 shows the causal structure of risk factors in the NHANES dataset. The structure was obtained using the proposed approach, which normalizes the raw data in the first step. Six causal relationships are shown in in Fig. 8. ${X_4} \rightarrow {X_5}$, ${X_4} \rightarrow {X_7}$, and ${X_4} \rightarrow {X_8}$ imply that body weight results in changes in BMI, creatinine in urine, and high-density lipoprotein, respectively; ${X_5} \rightarrow {X_7}$ indicates that BMI results in changes in creatinine in urine, ${X_9} \rightarrow {X_8}$ indicates that triglycerides result in changes in high-density lipoprotein, and ${X_{13}} \rightarrow {X_{11}}$ indicates that fasting glucose results in changes in glycated hemoglobin.

**Fig. 9:**
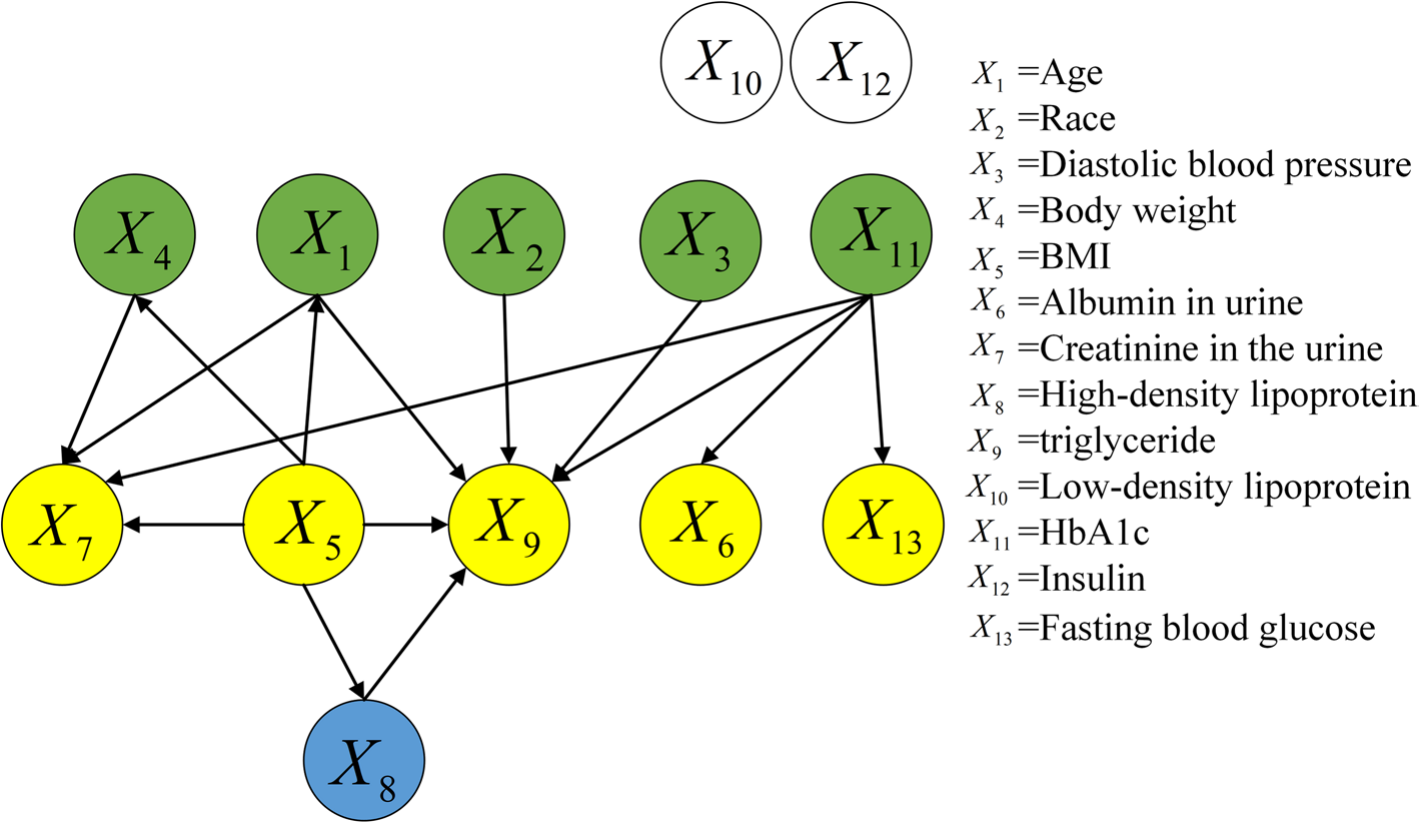
Causal structure of NHANES dataset (raw data)

Figure 9 presents the causal structure of risk factors in the NHANES dataset without normalization. The causal strengths are summarized in Table 1. Compared with Figs. 8 and 9 shows a few wrong relationships, in addition to the causal relationships mentioned in the preceding paragraph. For example, ${X_5} \rightarrow {X_1}$ indicates that BMI causes a change in age, which is neither sensible nor supported by any previous research. Additionally, ${X_5} \rightarrow {X_4}$ implies that BMI causes a change in weight. The correct causal relationship should be the opposite, i.e., a change in weight causes a change in BMI. The reason is straightforward: BMI, which is a function of body height and weight, cannot determine a person’s weight.

In general, when the raw data of the NHANES dataset were utilized, the causal structure obtained via causal discovery showed incorrect causal relationships. After performing data normalization, the causal structure became significantly simplified, and the correct rate improved considerably. Similarly, this proves that the accuracy of causal discovery can be effectively improved by normalizing the raw dataset in advance.

**Table 1:** Causal strengths of NHANES raw data

Causal relationship	Causal strength	Causal relationship	Causal strength
${X_1} \rightarrow {X_7}$	7.957	${X_5} \rightarrow {X_8}$	9.262
${X_1} \rightarrow {X_9}$	10.349	${X_5} \rightarrow {X_9}$	49.522
${X_2} \rightarrow {X_9}$	8.986	${X_8} \rightarrow {X_9}$	11.393
${X_3} \rightarrow {X_9}$	10.593	${X_{11}} \rightarrow {X_6}$	1.77
${X_4} \rightarrow {X_7}$	3.318	${X_{11}} \rightarrow {X_7}$	8.226
${X_5} \rightarrow {X_1}$	8.56	${X_{11}} \rightarrow {X_9}$	10.809
${X_5} \rightarrow {X_4}$	3.223	${X_{11}} \rightarrow {X_{13}}$	19.142
${X_5} \rightarrow {X_6}$	112.842		

## Discussion

The discussion part will demonstrate in detail each pair of causal relationship in the causal structure, explain the meaning of causal relationship, and analyze the relevant literature to discuss the correctness of causal relationship.

### Pima Indian diabetes datasets

An identical causal relationship is indicated in Figs. 4 and 5, namely, ${X_5} \rightarrow {X_2}$. This relationship is well known to the public, and studies have also shown [30, 31] insulin is a hormone that controls blood sugar in the human body and affects changes in blood sugar concentration. This causal relationship is clearly established.

Compared with Fig. 4 and 5 shows two causal relationships with a change in direction: ${X_6} \rightarrow {X_4}$ and ${X_8} \rightarrow {X_1}$. Between them, ${X_6} \rightarrow {X_4}$ indicates that the change in BMI results in a change in the skin fold thickness of triceps (subcutaneous fat thickness).Studies have shown [32, 33] that there is a significant correlation between the thickness of the triceps skin fold and BMI. When the BMI value increases, the weight change increases, and the subcutaneous fat thickness increases, so the causality is reasonable. ${X_8} \rightarrow {X_1}$ suggests that a change in age results in changes in gravidity. Based on common perception, more pregnancies are likely to occur in older people. By contrast, ${X_1} \rightarrow {X_8}$ in Fig. 4 indicates that gravidity affects age. Study [34] has shown that more pregnancies increase the physiological age and causes the cells to age faster. However, the age variable in the datasets is the actual age, not the physiological age. Although ${X_1} \rightarrow {X_8}$ presents a certain degree of reasonability, is more consistent with the real-world causal relationship, after considering the causal strength.

Figure 5 presents an additional causal relationship compared with Fig. 4, i.e., ${X_8} \rightarrow {X_3}$. This relationship indicates that a change in age alters the diastolic blood pressure, which is consistent with the medical law [35, 36] that blood pressure in general increases with age, since blood vessels become less elastic with age. Therefore, this causal relationship is reasonable.

### NHANES dataset

First, ${X_4} \rightarrow {X_5}$ implies that weight affects BMI. This is reasonable, as a change in weight alters the BMI because the latter is calculated based on height and weight. Thus, this causal relationship is correct.

Second, ${X_4} \rightarrow {X_7}$ and ${X_5} \rightarrow {X_7}$ indicate that creatinine in urine may vary with body weight and BMI, respectively. When a person gains weight, his/her muscle metabolism increases. This implies that an obese person may experience elevated creatinine.It was also shown [37] that urinary creatinine was a significant covariate of urine pH, which was negatively correlated with body weight in patients with stones. Therefore, these two causal relationships may be valid.

Third, ${X_4} \rightarrow {X_8}$ and ${X_9} \rightarrow {X_8}$ signify that high-density lipoprotein may vary with body weight and triglyceride level, respectively. Abnormalities in lipid metabolism caused by obesity are primarily manifested [38, 39] as hypertriglyceridemia, reduced high-density lipoprotein cholesterol, and increased small and dense low-density lipoprotein cholesterol. Hence, weight may cause abnormal changes in lipid metabolism, although further medical verification is necessitated.

Fourth, ${X_{13}} \rightarrow {X_{11}}$ indicates that fasting blood glucose affects glycated hemoglobin, which is the product [40, 41] of a non-enzymatic reaction combining hemoglobin with blood glucose. When a patient’s fasting glucose or postprandial glucose is not controlled well, the glycated hemoglobin will not satisfy the standard, which is manifested by an increase in his/her fasting blood glucose. This is generally accompanied by a significant increase in glycated hemoglobin. Therefore, this causal relationship is correct.

## Conclusions

In this study, a causal discovery approach with reinforcement learning for T2DM risk factors was proposed, through reinforcement learning model construction, design of causal discovery algorithm process and experimental verification analysis, the effectiveness and adaptability of this method are confirmed, and it has great potential in causal discovery of disease risk factors, it can provide a new attempt for diabetes prevention and research. The reinforcement learning model shall be improved in the future, which will include more clinical data analysis such that the ability of the proposed algorithm in mining and intuitively analyzing causal relationships can be further enhanced.

## Data Availability

The datasets generated and/or analysed during the current study are available in the [github] repository, [https://github.com/JGcuzme/RL].

## Abbreviations

T2DM: Type 2 diabetes mellitus
SD: Severe depression
PED: Perceived ethnic discrimination
IIE: Inverse information entropy
BIC: Bayesian information criterion
NHANES: National Health and Nutrition Examination Survey
BMI: Body mass index
HbA1c: Glycosylated hemoglobin
